# Recent Progress of Electrospun Nanofiber Dressing in the Promotion of Wound Healing

**DOI:** 10.3390/polym16182596

**Published:** 2024-09-13

**Authors:** Xiaoqi Lu, Libo Zhou, Weiye Song

**Affiliations:** 1 School of Mechanical Engineering, Shandong University, Jinan 250061, China; 2 School of Pharmaceutical Sciences, Cheeloo College of Medicine, Shandong University, Jinan 250012, China

**Keywords:** electrospun, wound healing, nanofiber, polymer

## Abstract

The nanofiber materials of three-dimensional spatial structure synthesized by electrospun have the characteristics of high porosity, high specific surface area, and high similarity to the natural extracellular matrix (ECM) of the human body. These are beneficial for absorbing wound exudate, effectively blocking the invasion of external bacteria, and promoting cell respiration and proliferation, which provides an ideal microenvironment for wound healing. Moreover, electrospun nanofiber dressings can flexibly load drugs according to the condition of the wound, further promoting wound healing. Recently, electrospun nanofiber materials have shown promising application prospects as medical dressings in clinical. Based on current research, this article reviewed the development history of wound dressings and the principles of electrospun technology. Subsequently, based on the types of base material, polymer-based electrospun nanofiber dressing and electrospun nanofiber dressing containing drug-releasing factors were discussed. Furthermore, the application of electrospun nanofiber dressing on skin tissue is highlighted. This review aims to provide a detailed overview of the current research on electrospun nanomaterials for wound healing, addressing challenges and suggesting future research directions to advance the field of electrospun dressings in wound healing.

## 1. Introduction

Skin, the largest organ of the human body, has the functions of blocking the invasion of foreign pathogens and preventing the loss of body fluids [1]. Skin is not only an important protective barrier for the human body but also an important part of the immune system. Local skin tissue is damaged due to instrument trauma, burns and pathological ulcers, resulting in the loss of its original function. Once harmful microorganisms invade the wound surface, they will cause infection, delay wound healing and even affect the normal life activities of the human body [2]. According to WHO, an estimated 180,000 deaths every year are caused by burns, which vast majority occur in low- and middle-income countries [3]. Globally, diabetic foot ulcers affect about 20 million people every year. Diabetic foot ulcers are the main cause of disability and death [4,5]. The process of wound repair is long and complex, which imposes a huge burden on patients and society. Therefore, ideal wound dressings need to be developed to replace damaged skin as a temporary barrier, avoiding or managing wound infection, providing a nurturing atmosphere that promotes wound healing, shortening wound repair time and promoting wound repair [6]. At present, wound dressings can be divided into traditional dressings and new medical dressings.

Traditional dressings such as gauze and cotton pads can only temporarily cover and protect the wound. However, the hemostasis effect of traditional dressings is not satisfactory. Then, they tend to adhere to the wound site, which can lead to secondary damage when the dressing is removed [7]. Traditional dressings cannot control the humidity of the wound microenvironment, which makes it difficult to inhibit the invasion of pathogenic microorganisms in the process of long-term use and increase the risk of wound infection. Currently, traditional dressings can no longer meet people’s needs for wound treatment [8].

The new medical dressing significantly overcomes the disadvantage of traditional dressings by ensuring local moisture for the wound, maintaining an oxygen environment in the local microcirculation of the wound, facilitating the dissolution of necrotic tissue, and promoting the proliferation as well as differentiation of cells [9]. The new medical dressing retains the active substances in the exudate and promotes their release, creating a healing environment that closely resembles the physiological state. Research shows that the new wound dressing effectively promotes wound healing, significantly reduces the adverse effects of inflammatory response, and alleviates patients’ suffering [10,11]. At present, new medical dressings mainly include hydrocolloids [12], alginate [13], hydrogels [14], synthetic fibers [15], and more. Among these, the nanofiber materials synthesized by electrospun technology are characterized by high porosity, large specific surface area and a high degree of similarity to the human body’s natural extracellular matrix, which provides an ideal platform for various tissue regeneration applications [16]. Electrospun nanofiber dressings are capable of absorbing wound exudate and effectively blocking the external environment, thereby facilitating wound healing. At the same time, electrospun can not only selectively load appropriate drugs according to different wounds to achieve functional enhancement but also does not affect the drug activity with the high voltage in the preparation process of electrospun nanofiber dressing [17]. Electrospun nanofiber dressing has broad prospects in the field of promoting wound repair.

Electrospun is a process for producing nanofiber materials. Under a strong electric field, the polymer solution or melt droplets transform from spherical to conical shapes, extending from the cone’s tip to form fibers with a nano-sized diameter [16]. Electrospun technology has attracted the attention of researchers due to its simple operation, the wide variety of raw materials available for spinning, and controllable processes [18]. At present, the preparation of new wound dressings using electrospun technology is in a rapid development stage. Due to the influence of factors such as spinning raw materials, spinning process, and spinning environment, the application of the final products is mostly still in the experimental stage and has not yet achieved large-scale industrial production. This article summarized and analyzed the performance characteristics of nanofiber wound dressings prepared by electrospun technology with different polymers and additives and discussed the application cases and research progress of electrospun nanofiber materials in promoting wound healing. Finally, the challenges in the research of electrospun nanofiber wound dressing were pointed out, which provided references for promoting the functional development and application of electrospun nanofiber materials in wound healing.

## 2. The Stages of Wound Healing

**Figure 1:**
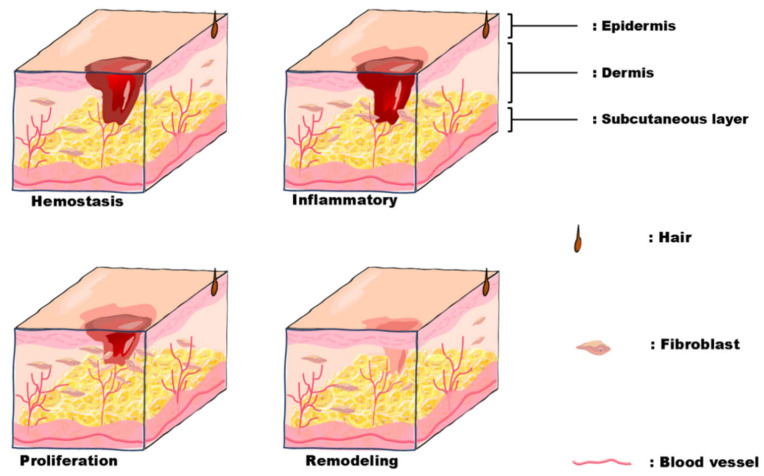
Wound healing process.

Skin is the largest organ of the human body, which makes direct contact with the external environment and plays a role in protecting, excreting, regulating body temperature, and feeling external stimuli [19]. The normal tissue structure of the skin can be damaged by sharp tools, burns, and other pathological factors and result in wounds [20]. The wound healing process mainly includes four phases, shown in Figure 1: the hemostasis phase, inflammatory phase, cell proliferation phase and tissue remodeling phase. It is a complex repair process in which cells at different stages cooperate.

Hemostasis phase [21]: Prevent massive bleeding after further vascular damage. Usually, in the first stage, coagulation and hemostasis occur within a few hours. After vasoconstriction, platelets and inflammatory cells initiate primary hemostasis at the site of injury, while platelets secrete a variety of growth factors. These growth factors facilitate the recruitment of epithelial cells and other cells, laying the groundwork for the inflammatory phase.

Inflammatory phase [22]: Early changes in wounds. The inflammatory phase follows the initial vasoconstriction at the onset of the hemostasis phase. The inflammatory phase usually lasts from 48 to 72 h, but it may also take 5 to 7 days to completely end. There are varying degrees of tissue necrosis and vascular rupture with bleeding locally within the wound. Due to vascular dilation and increased permeability, fibrin and growth factors derived from plasma leak out, facilitating the infiltration of inflammatory cells into the blood vessels. Within hours, an inflammatory response ensues, ultimately resulting in tissue fluid accumulation within the wound and swelling at the wound site.

Cell proliferation phase [23]: After 2–3 days, the entire layer of skin and subcutaneous tissue at the wound’s edge migrates towards the center so that the wound rapidly shrinks. During this phase, the release of various growth factors primarily stimulates cell proliferation. At the same time, the influence of the ECM facilitates cell differentiation, subsequently promoting tissue granulation, angiogenesis, and wound contraction. The cell proliferation phase will last for several weeks.

Tissue remodeling phase [24]: At this phase, the collagen of the wound gradually reorganizes and remodels to form new skin with enhanced strength and elasticity. Simultaneously, the new blood vessels further develop and mature to maintain the skin’s normal physiological function. The duration of the remodeling phase varies based on individual differences and the size of the wound, usually taking several months or even longer.

Proper management of skin injuries can help to accelerate the process of tissue repair and minimize the occurrence of complications. As a result, wound dressings were invented and have been continuously evolving [25].

## 3. Development of Wound Dressings

The main role of wound dressing in wound repair is to cover the damaged skin, maintain a moist environment and appropriate temperature, provide physical and microbial protection, absorb excessive tissue fluid, and relieve pain [26]. Traditional dressings are mainly used to directly cover mechanical injuries or wounds, using a honey paste, animal fat, plant fiber, and other materials, which serve as a barrier to the external environment. Because the drugs loading in traditional dressings typically are physical adsorption, the release time and effective time of drugs cannot be precisely controlled, thereby prolonging the wound healing time. In addition, traditional dressings are prone to adhere to the wound, potentially causing secondary damage during removal, and do not allow for good air permeability during wound healing. Therefore, traditional dressings are suitable for temporary treatment of simple, uninfected wound surfaces.

Different from traditional dressings, new medical dressings are developed on the basis of the moist healing theory. In 1962, British zoologist George Winter proposed the theory of moist healing, which states that a moderately moist and airtight environment is conducive to the rapid growth of epithelial cells and wound healing [27]. According to the mechanism of wound healing, an ideal wound dressing should be able to absorb excess exudate, prevent microbial infection of the wound, maintain a moist healing environment at the wound site, and meet the criteria of being non-toxic, biocompatible, and biodegradable [9]. With ongoing research into the wound healing process and advancements in material technology, new medical dressings now mainly include hydrocolloid dressings, alginate dressings, hydrogel dressings, synthetic nanofiber dressings, and more. Hydrocolloid dressings can maintain temperature, create a hypoxic environment, and provide a moist healing environment. However, the loss of adhesion in hydrocolloid wound dressings is a prevalent clinical issue [28]. Repeated replacements can harm the surrounding delicate skin, severely compromising treatment efficiency and patient experience [12]. Alginate is a popular biomaterial. Alginate dressings can absorb a large amount of exudate from the wound, hydrate with necrotic tissue, and aid in self-digesting debridement [13]. However, due to its fibrous nature, it should not be used for wounds with a small amount of exudation and eschar, as it may leave excess fibers in the wound and trigger inflammation [29]. The hydrogel dressing’s inherent water-containing property can provide a moist environment for the wound and create favorable conditions for tissue regeneration, making it suitable for eschar debridement [14]. However, fluid accumulation may also lead to wound infection and bacterial growth. Synthetic nanofiber dressings have high biocompatibility, exhibit similarity to the ECM, and are frequently utilized in the pharmaceutical and biomedical sectors [30]. In addition, synthetic nanofiber dressings also have biodegradable and bioabsorbable properties, which can promote the reconstruction of new tissues without causing inflammation.

The fibers prepared by dry spinning, wet spinning, and gel spinning methods rarely achieve the submicron level, but electrospun can easily obtain continuous and slender nano-sized fibers due to its unique process technology. Compared with traditional dressings, nanofiber dressings synthesized by electrospun technology possess outstanding characteristics such as high porosity, high specific surface area, high similarity to the natural ECM of the human body, and controlling drug release [31]. Electrospun nanofiber dressings are more conducive to wound tissue repair. In addition, strong electric fields will not affect the activity of drugs in the process of synthesizing electrospun nanofiber materials. By utilizing suitable materials as carriers and incorporating functional loads such as antimicrobials, growth factors, vitamins, and proteins, the wound repair capabilities of electrospun nanofiber dressings are further enhanced.

## 4. Principle of Electrospun

Electrospun is a production technology that can continuously produce nanofiber materials. As early as the 16th century, Gilbert found that after the solution was electrified, the droplets suspended in the solution transformed from circular to conical under the influence of an external electric field, which was the first time that the concept of electrospun was proposed [32]. In 1915, Taylor applied mathematical modeling to describe the process of charged polymer solution or polymer melt from circular to conical, which proved that the electric field force causes the charged droplet to stretch and deform. When reaching the critical tension of the droplet, the droplet is conical and shoots a straight jet at the apex of the cone. This conical droplet is called “Taylor cone” [33]. At the beginning of this century, the focus on electrospun has broadened significantly, with more new materials being utilized in the fabrication of ceramic and composite nanofibers. Concurrently, innovative advancements in electrospun technology have led to the widespread application of electrospun nanofiber materials, particularly in the field of wound dressing, where research has seen remarkable progress over the past two decades.

**Figure 2:**
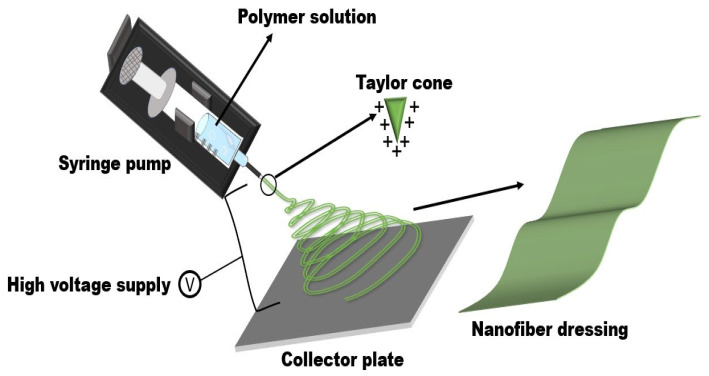
Schematic diagram of electrospun principle.

The principle of electrospun, as shown in Figure 2, involves applying a high voltage between the needle and the collector plate. As the voltage increases, the hemispherical droplets at the needle mouth experience a growing electric field force, leading to the formation of a “Taylor cone” [34]. This eventually overcomes the surface tension, causing the droplets to fall. As the solvent evaporates, the polymer solidifies, transforming from droplets into nanofiber materials.

The following are several common electrospun methods.

Co-electrospun: Co-electrospun involves mixing two or more polymer solutions into a homogeneous solution for electrospun to obtain multi-component nanofibers, which makes electrospun nanofiber materials have better morphology and wider applications. Feng Yunbo [35] prepared a novel multi-component zwitterionic gradient wound dressing using the co-electrospun method, achieving low biological adhesion, good humidity control, and long-term antibacterial performance during the healing process of chronic wounds [36]

Coaxial electrospun: Coaxial electrospun employs coaxial needles with inner and outer layers, which are respectively injected with a core solution and a shell solution. The two solutions are then extruded through the needles at a constant flow rate, resulting in the preparation of composite nanofibers with a ‘core-shell’ structure. Compared to traditional methods, coaxial electrospun offers enhanced stability. Furthermore, it can also utilize polymers as the ‘shell’ to serve as templates for substances that cannot be spun into fibers alone (such as proteins, drugs, etc.), thereby broadening its range of applicability. Majid Salehi et al. [37] used coaxial electrospun technology to load kaolin into an electrospun polyvinyl alcohol/chitosan polymer mixture to develop a composite nanofiber dressing for melanoma postoperative treatment. This nanofiber dressing not only possesses the capability for the sustained release of chemotherapy drugs but also promotes wound healing, offering a novel and effective approach for the postoperative treatment of melanoma [38].

Emulsion electrospun: Under the action of an emulsifier, two immiscible solutions are emulsified into emulsions, and “core-shell” structured fibers are directly obtained through spinning using a single needle. The two most common types of emulsions are oil-in-water (O/W) and water-in-oil (W/O). Compared with coaxial electrospun, this technique significantly simplifies equipment complexity and operability while also enhancing efficiency. Basar, A. O. et al. [39] mixed a poly(ε-caprolactone) (PCL) solution containing ketoprofen as the oil phase with gelatin dissolved in acidified water in a 7:3 (wt/wt). The obtained water-in-oil (O/W) emulsion stabilized by the PCL–gelatin complex (also containing ketoprofen at 5 wt%) was electrospun to prepare a binary wound dressing, demonstrating a sustained drug release capability of up to 4 days [40].

## 5. Types of Electrospun Nanofiber Dressings

The important application of electrospun nanofiber materials in the biomedical field is medical dressing [41]. The high specific surface area and high porosity of electrospun nanofiber dressing contribute to the absorption of wound exudates and drug release, ensure gas exchange, and effectively isolate the invasion of external liquids and bacteria, which provide an ideal microenvironment for promoting cell respiration and wound healing. Moreover, the flexible and convenient electrospun technology incorporates antibacterial agents, vitamins, and growth factors, among others, according to the actual situation of the wound, to further promote wound healing. In order to meet the actual demand, the release rate of drugs loaded on nanofiber materials is regulated by selecting different types of polymers, controlling the position of drugs within the nanofiber materials, and altering the drug content [42,43].

### 5.1. Polymer-Based Electrospun Nanofiber Dressing

#### 5.1.1. Natural Polymer-Based Electrospun Nanofiber Dressing

Natural polymers, such as polysaccharides, chitosan, and proteins, have antibacterial and hemostasis functions [44]. Natural polymers are synthesized into nanofiber dressings by electrospun technology, which can better promote cell adsorption and propagation and accelerate wound healing [45]. Hajali, Hadi et al. [46] demonstrated that the nanofiber dressings, which combine the natural polymer sodium alginate with lavender essential oil, offer advantages in reducing infection risk and controlling inflammatory response, proving their effectiveness in burn treatment. Natural polymer-based electrospun nanofiber dressing possesses excellent biocompatibility, degradability, low antigenicity, and strong antibacterial and hemostasis properties, which can promote wound healing. They are primarily utilized in tissue engineering and postoperative wounds. However, natural polymers have complex components and limitations of poor mechanical properties. At present, the spinnability is mostly improved by adding some substances with high mechanical strength [47].

#### 5.1.2. Synthetic Polymer-Based Electrospun Nanofiber Dressing

Synthetic polymers such as polylactic acid (PLA), polyvinyl alcohol (PVA), polycaprolactone (PCL) and more have excellent mechanical properties, thermal stability, and processing flexibility, making them widely used [48,49,50]. Blanquer Andreu’s [51] team produced nanofiber dressings composed of PCL and PVA layers rich in platelet lysates using a needle-free multi-jet electrospun method, which enabled cell adhesion and growth and was utilized as a continuous release system for bioactive compounds. However, synthetic polymers lack cellular binding sites and are non-biodegradable, which limits their effectiveness in promoting wound healing [16].

#### 5.1.3. Composite Materials

Electrospun wound dressings prepared from a single raw material are insufficient to meet the demands of wound care. To enhance the functionality of these dressings, researchers frequently combine natural polymers with synthetic polymers in the preparation of electrospun dressings, aiming to provide a more conducive microenvironment for accelerated wound healing [52,53]. Fatemeh Amini et al. [54] prepared poly-hydroxybutyrate/chitosan nanofibers loaded with gentamicin and attached a layer of polyvinylidene fluoride by electrospun, which enabled controlled release of the drug and enhanced mechanical strength, making it suitable for use as a wound dressing for postoperative ulcers.

### 5.2. Electrospun Nanofiber Dressing Containing Drug-Releasing Factors

Although natural polymers and synthetic polymers possess numerous excellent properties, their capacity to promote wound healing is limited. Nanofiber materials are utilized as the substrate of dressings, loaded with drug factors, enabling them to fulfill their roles during various stages of wound healing and promote wound repair [52]. Electrospun nanofiber dressings containing drug factors mainly add drugs or growth factors with antibacterial and hemostasis properties into the electrospun solution, thereby enhancing the performance of wound excipients and facilitating wound healing [55,56].

#### 5.2.1. Adding Antimicrobial Agents

Bacterial infections often lead to an inflammatory response and delay wound healing [57]. Silver ions have strong bactericidal and antibacterial effects [58,59]. As a type of new medical dressing, silver (Ag) nano dressing can release Ag during the treatment of wound healing, which effectively prevents wound cross-infection and promotes wound healing [60]. Samanta, A. et al. [61] synthesized high-yield nano silver in an aqueous medium using silver nitrate (AgNO_3_) as the precursor, with sodium borohydride (NaBH_4_) and polyethylene oxide (PEO) serving as the dispersed phase of the emulsion. The continuous phase of the emulsion was composed of the PCL solution in toluene. They varied the concentrations of AgNO_3_ and PCL to investigate their impact on the emulsion’s stability. In comparison to the emulsion formed using NaBH_4_, the nano silver produced with PEO as the reducing agent resulted in a highly stable emulsion. The in situ synthesized silver nanoparticles were utilized as Pickering stabilizers for the poly(ε-caprolactone) (PCL) water-in-oil emulsion. The resulting dressing exhibited uniformity in its fiber matrix, which contained silver nanoparticles and demonstrated antibacterial properties. Ag dressing is mainly applied to open wounds, which can absorb exudates, effectively inhibit bacterial reproduction and promote wound healing [62]. Currently, most Ag dressings available in the market have shortcomings in limited antibacterial duration, frequent dressing changes, high cost, and controversial toxicity in wound healing [63,64].

#### 5.2.2. Adding Antibiotics

Antibiotics have become the first choice for the preparation of antimicrobial electrospun wound dressing because of their good antimicrobial properties. The antimicrobial dressing is prepared by electrospun the polymer solution containing antibiotics, which can enlarge the surface area of the dressing that is insoluble in water or not easily absorbed by the human body. This makes the antibiotics gradually diffuse into the human body with the fibers degrade, facilitating the absorption by human tissues, prolonging the drug release duration, and enhancing the efficacy of the drug. Therefore, nanofiber dressings containing antibiotics are widely used in drug wound repair. Liu Shuiping et al. [65] prepared coaxial nanofiber mats with poly(L-lactyl-co-ε-caprolactone) (PLLACL) as the shell and Tetracycline Hydrochloride (TCH) as the core through coaxial electrospun technology. In the in vitro environment at 37 °C, the release duration of TCH reached up to 180 h, achieving effective encapsulation of TCH within the (PLLACL) nanofibers, ensuring a prolonged drug release effect. In addition, researchers have also utilized antibiotics such as cefazolin [66] and ofloxacin [67] as antimicrobial agents for electrospun nanofiber dressings, all of which exhibited good antimicrobial effects. Antibiotic dressings possess excellent antimicrobial properties, however, with the increasing resistance of bacteria to antimicrobial agents, especially antibiotics, there is an urgent need to develop new dressing that can effectively inhibit pathogenic microorganisms.

#### 5.2.3. Adding Animal and Plant Extracts

**Figure 3:**
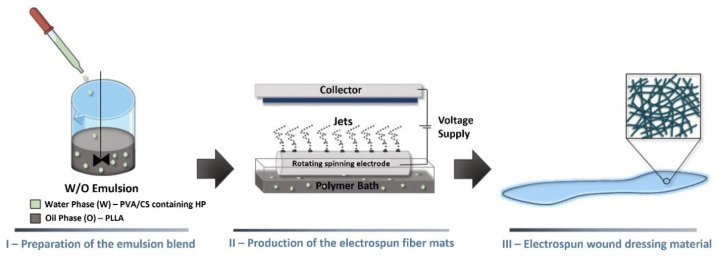
Preparation process of PLLA/PVA/CS wound dressing by W/O emulsion electrospun. Reproduced from [71] MDPI 2023.

Numerous studies have shown that animal and plant extracts, as natural active substances, possess excellent antimicrobial ability to promote wound healing. Aloe vera gel applied to the burned area can promote skin healing and provide relief [68,69]. Consequently, the development of electrospun wound dressings incorporating animal and plant extracts has emerged as a research hotspot in recent years [46,70]. Cláudia Mouro et al. [71] developed a poly(L-lactic acid) (PLLA)/poly(vinyl alcohol) (PVA)/chitosan (CS) wound dressing material through W/O emulsion electrospun, shown in Figure 3, which is produced in a single step using uniform gel-like suspensions of two different and incompatible polymer solutions. The electrospun PLLA/PVA/CS fiber mats were loaded with two different amounts of Hypericum perforatum (HP) (2.5% and 5.0%). The electrospun PLLA/PVA/CS fiber mats containing 2.5% HP can prevent the growth of the Gram-positive bacterium Staphylococcus aureus without causing cytotoxicity to normal human dermal fibroblasts, contributing to the prevention of wound infections and providing appropriate support and a microenvironment for wound healing. Herbs, as traditional medicine, have been utilized for many years in many countries such as China and India. The effective components extracted from them can address the shortcomings of inorganic antimicrobial dressings, particularly with regard to water solubility and cytotoxicity. The application value of herbs in antimicrobial dressings still requires further research.

#### 5.2.4. Adding Growth Factors

In the stage of granulation tissue reconstruction and remodeling, drugs that can accelerate cell tissue migration and promote cell regeneration are needed. These drugs, when added to wound dressings, can shorten wound healing time and improve wound healing rate [72]. Many researchers incorporate growth factors as active substances into wound dressings, thereby promoting wound healing and enhancing performance [20]. A. Schneider [73] functionalized the electrospun nanofiber pad by adding an epidermal growth factor, which gradually released over time and increased the wound closure rate by more than 3.5 times. Zhang et al. [74] used the emulsion electrospun method to prepare basic fibroblast growth factor sustained-release nano dressings with active wound healing properties from adenosine triphosphate or its salt and metal ion emulsions, which include basic fibroblast growth factor and electrospun nanofiber dressings.

## 6. Research Progress of Electrospun Nanofiber Dressings on Skin Tissue

Skin is often directly exposed to adverse environmental factors (such as harmful pathogens, physical hazards, and chemicals), making it susceptible to damage [75,76]. Typically, wounds undergo several consecutive and overlapping stages of cellular and biochemical activities to heal. Based on the healing time, wounds can be divided into acute wounds and chronic wounds [77], including surgical wounds, diabetic foot ulcers, and more. As a global medical issue, wound repair faces significant clinical challenges [78]. In order to meet clinical needs, new multifunctional medical dressings are developing rapidly. In recent years, nanofiber materials prepared by electrospun possess porous structure and high porosity, offering hemostasis and absorbent properties [79]. Moreover, these electrospun nanofiber materials can simulate the microstructure of ECM, capable of promoting cell migration and proliferation, thereby holding great promise for application in the field of wound dressing [80].

### 6.1. Acute Wound

An acute wound refers to a wound that suddenly forms and heals quickly or a wound that can heal within the expected time without complications after proper treatment [81]. During the normal healing process of a wound, if it is affected by certain factors such as ischemia, infection, dryness, etc., the wound healing can be delayed or even stalled, resulting in a chronic wound that is difficult to heal [82].

#### 6.1.1. Surgical Wound

Most surgeries leave a wound with its edges sewn together using sutures, staples, clips, or glue [83,84]. Postoperative wound infections are a common occurrence and can lead to pain, delayed wound healing, the need for long-term antibiotic use, prolonged hospital stays, and increased economic burden [85]. Proper selection of postoperative dressings can provide a moist wound environment, reduce the risk of infection and blistering at the surgical site, and promote effective wound healing. At present, a variety of different electrospun nanofiber materials have been used in the treatment of surgical wounds [18,86]. Lowe, A. et al. [87] used electrospun to incorporate acrylonitrile-based ternary copolymers and NO into the main chain polymer by forming azo-dicarboxylic acid groups, thereby producing nanofiber dressings. This study created a single excised wound with an average diameter of 0.50 cm^2^ on the back of mice to observe the release of NO dressing. The NO dressings were applied to the wounds, which demonstrated that delivery of NO with a novel bandage accelerates wound healing through NO-induced angiogenesis. Kao et al. [88] prepared lidocaine/human epidermal growth factor embedded nanofiber membranes using coaxial electrospun technology for the treatment of postoperative pelvic adhesions. Abdominal surgery was performed on rats to simulate gynecological surgery in order to evaluate the biocompatibility and efficacy of antiadhesion membranes embedded with anesthetics/growth factors. Human epidermal growth factor participates in skin wound healing by stimulating, proliferating, and migrating keratinocytes, endothelial cells, and fibroblasts; it is beneficial for skin regeneration, and lidocaine can effectively relieve pain. Anesthetics and human epidermal growth factor nanofibers have great potential for the treatment of postoperative adhesions. But the main limitation of local anesthetics is systemic blood toxicity. Dressings should be scientifically loaded with anesthetics to ensure their safety for the human body.

#### 6.1.2. Burn Wound

**Figure 4:**
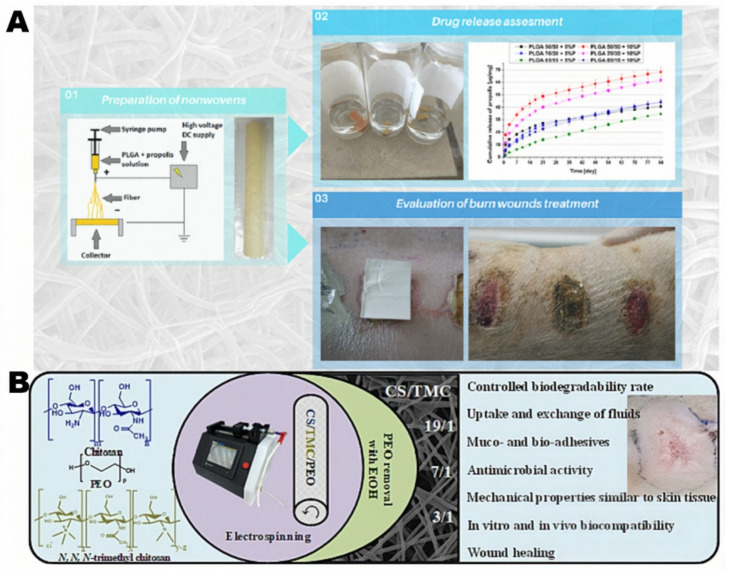
Preparation process of electrospun wound dressing of (**A**) PLGA and (**B**) chitosan/ TMC. (**A**) reproduced from [93] MDPI 2020. (**B**) Adapted with permission from [94] ELSEVIER 2023.

Burn wound encompasses skin damage resulting from exposure to heat, extreme cold, electrical currents, and chemicals, among others [89]. Due to the increase of oxygen-free radicals in burn wounds, the damage to vascular endothelial cells is exacerbated, resulting in ischemia and hypoxia in the wound, which slows down wound healing [90]. Additionally, the destruction of blood vessels and sweat glands in burn wounds complicates maintaining adequate local blood circulation and keeping the wound moist, further hindering healing [91]. Hence, burn dressings must ensure local blood circulation, facilitate wound debridement and resist bacterial invasion to prevent infection [45,92]. Given the severity of burn injuries and the urgency of treatment, the research and development of burn dressings are increasingly vital. Mateusz et al. [93] made a biodegradable wound dressing from poly(lactic-co-glycolic acid) (PLGA) and propolis solution by electrospun method, as shown in Figure 4A. They created wounds on the skin of domestic pigs through electrode heating to simulate clinical skin burns. On the day 21 in vivo experiment, four domestic pigs with third-degree burns did not need to administer active substances in the form of ointment or cream daily. The propolis released from the non-woven cloth promoted burn wound healing. Alexandru et al. [94] prepared fully biodegradable chitosan/N,N,N-trimethyl chitosan (TMC) nanofiber dressings using electrospun and poly(ethylene glycol) as a sacrificial additive, which is shown on Figure 4B. This dressing exhibits strong antibacterial activity, high swelling capacity, fluid exchange, biodegradability, and biocompatibility. The application in a rat deep burn model demonstrated wound closure and positive healing effects with complete restoration of the epidermis. Electrospun nanofiber materials can absorb exudates and isolate external bacteria, so they have broad prospects in the treatment of burn wounds.

### 6.2. Chronic Wounds

Chronic wounds are those that cannot fully heal within a short period of time, presenting a significant challenge to clinicians and patients [95]. Generally speaking, wounds that fail to heal completely within 4–8 weeks can be considered chronic. If wound healing stops during the inflammation or proliferation stage, a normal wound can turn into a chronic wound, leading to impaired wound cell function and failure of normal healing [96,97].

#### 6.2.1. Diabetic Foot Ulcer

**Figure 5:**
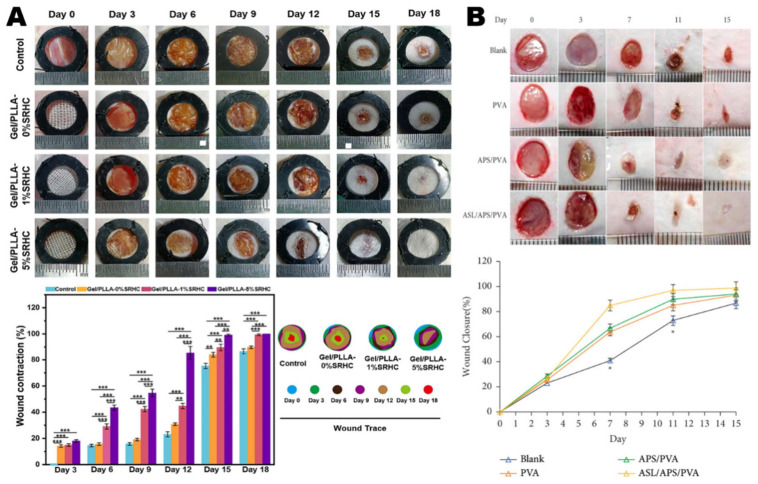
(**A**) Gel/PLLA-0% SRHC, Gel/PLLA-1% SRHC and Gel/PLLA-5% SRHC fabric dressing patches were used to treat the wounds of diabetic mice (** *p* < 0.01, *** *p* < 0.001). Adapted with permission from [104] ELSEVIER 2023. (**B**) The application of polyvinyl alcohol/ APS/ AS nanofiber membrane dressings using electrospun technology in the wound healing of diabetic rat. Reproduced from [105] WILEY 2022.

Diabetic foot ulcer (DFU) is a common and serious type of chronic wound in clinical settings [98]. In the clinical pathogenesis of DFU, peripheral neuropathy has a significant impact. Neuropathy of the lower extremities leads to loss of protective sensation, foot muscle atrophy, subcutaneous hemorrhage, and skin ulcers [99,100]. DFU management standard practices include wound off-loading, surgical debridement, blood glucose control, and dressings, which aim to promote a moist wound environment, control exudation, assess vascular function, and manage infection [101,102,103]. Choosing appropriate wound dressing can effectively promote wound healing and may reduce the incidence rate and economic burden on patients. Li et al. [104] prepared gelatin(Gel)/poly (lactic acid) (PLLA) nanofibrous loading with or without Salvia miltiorrhiza Bunge-Radix Puerariae herbal compound (SRHC) for wound treatment of diabetic patients through a modified electrospun strategy. The typically streptozotocin-induced diabetic mouse model with the full-thickness skin wound was created and employed to evaluate the biological functions of the Gel/PLLA nanofiber dressing. Macrophages usually secrete proinflammatory factors at the wound surface. The Gel/PLLA-0%SRHC nanofibers could inhibit the IL-6 secretion of LPS-induced M1 macrophage phenotype. The dressings with Gel/PLLA-5%SRHC could significantly shorten the healing time, and which wound closure rate reached 100% after 18 days of treatment. Yuan et al. [105] prepared polyvinyl alcohol/astragalus polysaccharide (APS)/astragaloside IV (AS) nanofiber membrane dressings using electrospun technology. In diabetic rat models displayed in Figure 5B, the nanofiber membrane dressings loaded with APS and AS exhibited significant wound repair capabilities, capable of inhibiting inflammation, enhancing collagen fiber deposition and regenerating epithelial repair, thereby significantly promoting the healing of diabetic wounds. Electrospun nanofiber materials hold great promise for wound dressings for diabetic wounds and tissue engineering.

#### 6.2.2. Chronic Leg Ulcer

**Figure 6:**
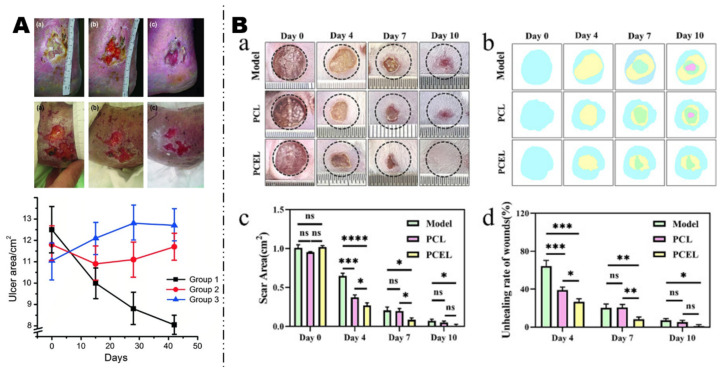
(**A**) Changes in the area of leg ulcers over time under using antibacterial dressing consisting mixed with a tetraphenylporphyrin photosensitizer by polyurethane techophilic^TM^ and electrospun. Adapted with permission from [106] WILEY 2012. (**B**) The electrospun polycaprolactone/chitosan/hEGF/lidocaine nanofibers dressing for the treatment of 2 stage pressure ulcers. (**a**) The images of wounds in the model group, PCL group and PCEL group on different days. (**b**) Image software simulates the process of wound healing. (**c**) Scar area. (**d**) Wound unhealing rate. (* *p* < 0.05, ** *p* < 0.01, *** *p* < 0.001, **** *p* < 0.0001, ns is no significant difference). Adapted with permission from [107] ELSEVIER 2024.

A leg ulcer affecting deep tissues is the most common type of chronic wounds, which bring a heavy economic burden to the health care system and lead to long-term suffering of patients. Monika et al. [106] conducted a study on a novel antibacterial dressing consisting of polyurethane techophilic^TM^, which was created through electrospun and mixed with a tetraphenylporphyrin photosensitizer that is activated by visible light. The prepared new antibacterial dressing was clinically applied to 162 patients with leg ulcers using a group comparison test who were treated with and without light or dressing. In 89 patients, wounds exhibited a reduction in size, decreased levels of exfoliated tissue and fibrin cover, and increased levels of healthy granulation tissue formation and epithelialization. Additionally, there was a reduction in wound-related pain. The study of Monika et al. demonstrates the exciting application of electrospun nanofiber dressing in leg ulcer wounds, which is shown in Figure 6A. However, tetraphenylporphyrin is a photosensitizer, which is irritating to the skin. For such dressings, skin irritation and other adverse effects should be more fully considered.

#### 6.2.3. Pressure Sore

A pressure sore is an ulcer caused by applying prolonged pressure to the tissue, usually located on bone prominences [108]. They are a common complication among hospitalized patients. Because pressure sore often cannot successfully go through the inflammatory stage of wound healing, current clinical adjunctive treatments mainly involve using dressings to provide moisture and absorb exudate, local application of biological agents, debridement surgery to remove necrotic tissue, and frequently rotating patients to alleviate pressure [109]. To minimize damage to the skin, protecting the wound site to reduce the risk of infection and accelerating the wound healing process is crucial. Dong et al. [107] prepared electrospun nanofiber dressings, which were combined with chitosan and polycaprolactone as scaffold materials and loaded with human epidermal growth factor (hEGF) and lidocaine using the ischemia-reperfusion method to simulate the stage 2 pressure ulcer wound of a human body on the surface of mice skin. Then, the prepared nanofiber dressing was applied to stage 2 pressure ulcers, shown in Figure 6B. In Dong’s research, hEGF was provided to promote cell proliferation, while chitosan exhibited significant antibacterial properties. Lidocaine, an analgesic, can be released immediately upon contact with the wound to reduce pain. The multifunctional electrospun nanofiber dressings offer an ideal auxiliary method for chronic wound healing and showcase the potential application of electrospun nanofiber dressing in the treatment of chronic wounds such as pressure ulcers.

## 7. Summary

Electrospun nanofiber materials with high porosity, high specific surface area, and micron-sized pore size can effectively prevent external bacteria and maintain a moist wound environment, thereby facilitating the absorption of wound exudate and providing a conducive microenvironment for wound healing. Additionally, electrospun nanofiber materials can easily be loaded with drugs, thereby shortening the wound-healing process. Currently, significant advancements have been made in the research of electrospun nanofiber dressings, but there are still some limitations. Firstly, the development of electrospun devices and the setting of spinning parameters significantly impact the diameter and performance of nanofiber materials, making it challenging to obtain high-quality nanofiber materials. Secondly, electrospun nanofiber dressings are predominantly prepared in laboratories, so the efficiency of industrial batch production needs improvement. Therefore, it is necessary to develop more advanced equipment to facilitate sustainable commercial and industrial operations. Furthermore, after loading the functional additives onto the nanofiber materials, it is crucial to minimize the residual organic solvents present in the nanofibers and transition to eco-friendly solvents or even solvent-free systems. This ensures that the prepared dressings are not only safer but also more environmentally friendly, thereby enabling a smoother transition from theoretical experiments to clinical practice.

## Data Availability

Not applicable.
